# Supplementary material for: Gene expression and soluble protein level of PD-1 and its ligands (PD-L1 and PD-L2) in endometrial cancer
Source: PLoS One. 2025 Mar 5;20(3):e0312765. doi: 10.1371/journal.pone.0312765 (PMC11882069; doi:10.1371/journal.pone.0312765)
Supplement: Attachment — Submitted filename: Response to Reviewers.docx [file pone.0312765.s001.docx]

Response to comments raised by Editors and Reviewers on manuscript PONE-D-24-25776 titled: Gene Expression and Soluble Protein Level of PD-1 and Its Ligands (PD-L1 and PD-L2) in Endometrial Cancer

**Dear Editor**

We appreciate the editors and reviewers for taking the time to review our manuscript PONE-D-24-25776 and the valuable comments. Below are our responses to the comments in numerical order. We made the corrections according to the suggestions and attached the main manuscript, both with track changes and clean copy.

Review Comments to the Author and Response

**Reviewer #1:**

*Present article is so interesting and must be valuable for the patients suffering from uterine endometrial cancer. However, several concerns remain in this article, which suggest as follows:*

Comment 1:

*How have you selected the healthy control patients and obtained the endometrial samples? Usually, over 55-years-old females have passed the final menstruation, which make so hard to collect the endometrium. Furthermore, the endometrium from the menopaused female contains relatively less glandular cells compared to pre-menopausal female, which is differ from the breast or lung tissue.*

Response 1:

Thank you for the comment. Details have been added as needed. For the control group, samples were collected from benign cases with no significant uterine or ovarian pathology detected on imaging. Endometrial sampling, performed using a pipelle, will be histologically confirmed as normal endometrium. This sentence has been added to the manuscript, lines 122-134. Regarding the age range of the control group, it was between 30 and 60 years.

Comment 2:

*As you mentioned in LL321-323, soluble PD-1 and PD-L2 level showed significantly differ age-dependent manner. Actually, these protein level seems associated with age in the ‘case group’, however, as you have not adjusted the age in the ‘control group’, it is impossible compared between two groups.*

Response 2:

All samples in the control group were under 60 years old, which precludes it from being considered a subgroup since the cancer group includes ages both below and above 60. The manuscript has been updated to include information about this limitation, specifically addressing the challenge of obtaining samples from individuals older than 60 for the control group (see lines 465-466).

Comment 3:

*In the process of RNA extraction from the endometrial tissue of control female, have you checked the infiltration of immune cells preliminary? The infiltration of immune cells in the uterine endometrium shows variety during the menstrual cycles in the pre-menopaused female, which may distort your data.*

Response 3:

Control samples collected are from women aged between 30 and 60 years old, with half of them being menopausal (see line 198-199). Before RNA extraction, the pathologist evaluated the tissue samples by performing H&E staining on a tissue section to inspect for the presence and distribution of immune cells visually (info added in text as necessary, in line 141-144).

**Reviewer #2:**

*Thank you for allowing me to review this paper. The paper investigate the role of PDL1 expression in ec and its role*

Comment 1:

*There is a strong correlation between pdl1 expression, TMB, and MMR status. It means that just having MMR status is useful to select patients who fit for ICI. Please comment on this.*

Response 1:

The necessary information has been added in discussion, as indicated in lines 444-450.

Comment 2:

*In the introduction of the paper is important to comment on the recent, cumulative data on ICI in first line setting (PMID: 38431043).*

Response 2:

Thank you for the suggestion. The necessary information has been added in introduction, as indicated in lines 91-104.

Comment 3:

*How about the role of molecular classification*

Response 3:

Thank you for the suggestion. The necessary information has been added in discussion as line 426-436.

Comment 4:

*How about PDL1 in multiple classsfier endometrial cancer. please discuss this point*

Response 4:

Thank you for the suggestion. The necessary information has been added in discussion, as indicated in line 437-443.

Comment 5:

*How about p53 wt and the potential role of selinexor (PMID: 37271639) in patients with and without Pdl1 expression*

Response 5:

Thank you for the suggestion. The necessary information has been added in line 451-461.

Thank you.

Best Regards,

Dr. Chew Kah Teik
